# Needs assessment of the advanced Ghana field epidemiology and laboratory training program, April 2024: lessons learned and best practices

**DOI:** 10.3389/fepid.2025.1646076

**Published:** 2025-12-02

**Authors:** Mariame Bonkano Laurent Comlan, Joseph Asamoah Frimpong, Charles Lwanga Noora, Donne Kofi Ameme, Aishat Bukola Usman, Virgil Kuassi Lokossou, Peter Evans Thomas, Danielle Thompson Barradas, Ditu Kazambu, Herbert Brian Kazoora, Ernest Kenu, Simon Nyovura Antara, Kerton Richard Victory

**Affiliations:** 1 Economic Community of West African States Regional Centre for Surveillance and Disease Control (ECOWAS-RCSDC), Abuja, Nigeria; 2 Ghana Field Epidemiology and Laboratory Training Programme (GFELTP), School of Public Health, University of Ghana, Accra, Ghana; 3 Division of Global Health Protection, Centers for Disease Control and Prevention (US-CDC), Atlanta, GA, United States; 4 African Field Epidemiology Network (AFENET), Kampala, Uganda

**Keywords:** FELTP, Ghana, workforce, one health, assessment

## Abstract

**Background:**

The Ghana Field Epidemiology and Laboratory Training Program (GFELTP) trains skilled field epidemiologists to strengthen surveillance systems and respond to public health threats. This assessment aimed to evaluate GFELTP's achievements, identify gaps in training and service delivery, and provide recommendations for improvement.

**Methods:**

A convergent mixed-methods evaluation was used, combining a self-administered questionnaire, program record reviews (graduation rates, surveillance outputs, publications), and three Focus Group Discussions (FGDs) involving staff, alumni, mentors, and residents. Thematic content analysis and triangulation with quantitative data were conducted to assess achievements and training gaps from 2007 to 2024.

**Results:**

Twenty-four participants (4 staff, 5 alumni, 5 mentors, and 10 residents) were interviewed. GFELTP operates as a regional program, training individuals from seven African countries. From October 2007 to March 2024, it enrolled 17 cohorts, producing 192 graduates and training 35 current residents. Of the graduates, 72% (139/192) were Ghanaians. Most graduates (89%) came from the human health sector, with 8% from the animal health sector and 3% from environmental health. Residents and graduates conducted over 200 outbreak investigations and evaluated more than 300 surveillance systems. They also delivered over 350 scientific presentations locally and internationally. FGDs revealed several challenges: limited mentorship funding, low program visibility, inadequate digital capacity for modern public health practices, and limited funding for resident exchange programs.

**Conclusions:**

GFELTP has made substantial contributions to public health capacity-building in Ghana and West Africa, through training, outbreak response, and scientific engagement. Key strengths include its regional reach, robust alumni network, One Health integration, and strategic collaborations. However, challenges remain in mentorship support, online visibility, and funding for resident development opportunities. Addressing these gaps through sustained mentorship, improved stakeholder engagement, and enhanced resource mobilization will further strengthen the program's impact and long-term sustainability in building a resilient public health workforce.

## 1 Introduction

The U.S. Centers for Disease Control and Prevention (CDC)'s Field Epidemiology and Laboratory Training Programs (FELTPs) are essential initiatives modeled after the CDC's Epidemic Intelligence Service (EIS) training (1, 2). FELTPs are integrated training and service programs that focus on building the capacity of public health professionals to perform critical field epidemiology practice, thereby strengthening public health systems. This initiative is one of the pillars of the Global Health Security Agenda (2). Since 1980, the CDC and partner organizations have assisted and supported Ministries of Health (MOHs) and other public health authorities in establishing FETPs in over 70 countries (2). The training is based on “learning by doing”, and trainees spend approximately 75% of their time developing their competencies in field epidemiology. There are three tiers of field epidemiology training: Frontline (3 months), Intermediate FETP (9 months), and Advanced FELTP (2 years) (3). Beyond focusing on human health, many FETPs also incorporate One Health principles, recognizing the interconnection between people, animals, plants, and their shared environment. These programs provide advanced training to health professionals, enabling them to respond effectively to public health emergencies, conduct disease surveillance, and implement prevention and control measures.

Field epidemiologists are essential in monitoring and analyzing health information, identifying and responding to disease outbreaks, investigating public health threats, and implementing evidence-based interventions. Laboratory scientists complement these efforts by providing accurate diagnostic services, supporting surveillance activities, and researching pathogens. Together, these professionals form the pillar of a robust public health response system. The primary objective of FELTPs is to establish a public health system that can rapidly identify and respond to health threats while also contributing to the development of scientific, evidence-based policies (4).

The Ghana Field Epidemiology and Laboratory Training Program (GFELTP) began in 2007 at the School of Public Health, University of Ghana. The GFELTP adheres to the general design principles of other FETPs but is tailored to the specific needs of Ghana and the West African region. Ghana is a founding member of the African Field Epidemiology Network (AFENET) and was the first program established in the West African subregion (5). The Advanced level of the GFELTP welcomes residents from several countries and has trained health officers from Liberia, Sierra Leone, The Gambia, Ghana, Zambia, Guinea-Bissau, and the Democratic Republic of Congo. The Training Programs in Epidemiology and Public Health Interventions Network (TEPHINET) accredited the program in 2016 and is preparing for reaccreditation. TEPHINET is a global network of Field Epidemiology Training Programs that strengthen public health capacity worldwide. For an FETP program, TEPHINET accreditation ensures adherence to international standards, enhances program credibility, and facilitates partnerships for resource mobilization, as seen in GFELTP's 2016 accreditation and ongoing reaccreditation efforts.

The curriculum combines 25% didactic sessions and 75% fieldwork. As TEPHINET recommends, residents spend 68 weeks in the field over two years. Since GFELTP was launched, an annual internal evaluation has been carried out every year. However, aside from the 2016 TEPHINET accreditation evaluation and assessment, the needs assessment described in this paper is only the second external evaluation carried out. External stakeholders promote a neutral and fresh perspective, making it possible to validate the internal assessment carried out by GFELTP. An external evaluation measures the reliability of the internal system established by the authorities and those responsible for implementing the program.

Evaluating FELTPs is crucial to ensure they achieve their objectives and contribute to enhancing public health services. Evaluation helps establish whether FETPs adequately equip trainees with the necessary skills to conduct field epidemiology tasks related to public health surveillance, outbreak investigation, and response. Evaluation feedback will help FETPs refine their approaches to mentorship, mobilizing funds, and addressing technological gaps. This fosters a dynamic learning environment responsive to emerging needs in public health. Demonstrating the impact of a program through evaluation ensures commitment from governments, partners, and funders. This is often justification enough to ensure continuous investment in FETPs through the various assessments capable of showing measurable feats to guarantee their long-term sustainability (6).

The GFELTP assessment aimed to identify gaps in training and service delivery, document lessons learned and best practices and provide recommendations for improving program effectiveness (7).

## 2 Methods

### 2.1 Study setting and population

This assessment was conducted from April to May 2024 and consisted of interviews with program staff, alumni, mentors, and residents of the advanced program. The program contacted participants via telephone and virtually scheduled interviews at mutually convenient times. We conveniently sampled and identified the GFELTP's current advanced residents, alumni, staff, and mentors based on their availability during the assessment period. We used the list of available residents, mentors, and alumni to select 10 participants from the resident's category and 10 from the mentors and alumni categories.

### 2.2 Data collection

We described the following from the program's inception to March 2024: number of residents and graduates; number of deliverables (e.g., surveillance system evaluations) completed by the trainees; number of partnerships with other programs in Africa and worldwide; number and frequency of internal evaluations conducted; types of mentorship strategies used, the MOH and University of Ghana roles, duration of advanced program relative to the CDC standard of 68 weeks of field activities; current academic and professional status of graduates. We also describe best practices, lessons learned, and challenges the program managers, staff, and resident's encounter. We provided a self-administered questionnaire to the GFELTP resident advisor to capture information on the context of the program implementation and operations, achievement, and best practices, such as exchange programs, curriculum adaptability, the One Health approach, and output-driven deliverables and advocacy for local ownership. The program documents were reviewed to triangulate the information from the self-administered questionnaire. Three focus group discussions (FGDs) among Program staff, mentors and alumni, and graduates and residents of the advanced program were conducted. Each session lasted three hours daily for over two days. A smartphone voice recorder was used to capture discussions, which were subsequently transcribed.

### 2.3 Data processing and analysis

We transcribed the qualitative data from notes and audio recordings and conducted a thematic analysis using an inductive approach, allowing key themes to emerge. The investigators coded the transcripts manually and compared findings to ensure inter-coder reliability. Discrepancies in coding were resolved through consensus discussions. Thematic saturation was determined when no new themes emerged after analyzing multiple focus group discussions. This process helped identify the best practices, lessons learned, challenges, program effectiveness and efficiency, resource mobilization and allocation, and inter-program exchange.

## 3 Ethical consideration

The assessment involved routine program monitoring data and did not include experimental procedures or direct human subject research. All data were handled with strict confidentiality protocols. Personal identifiers were removed prior to data analysis and securely stored in a separate, protected database to ensure participant anonymity.

## 4 Data availability statement

The original contributions presented in the assessment are included in a trip report submitted to GFELTP, WAHO-RCSDC and CDC on June 2024 and the FGD transcription summary. The data will be shared on reasonable request.

## 5 Findings

We identified 24 people with a 100% response rate; four (17%) were program staff, five (21%) served as mentors, five (21%) as alumni, and 10 (42%) were current trainees of the Advanced program.

### 5.1 Implementation successes

**Figure 1:**
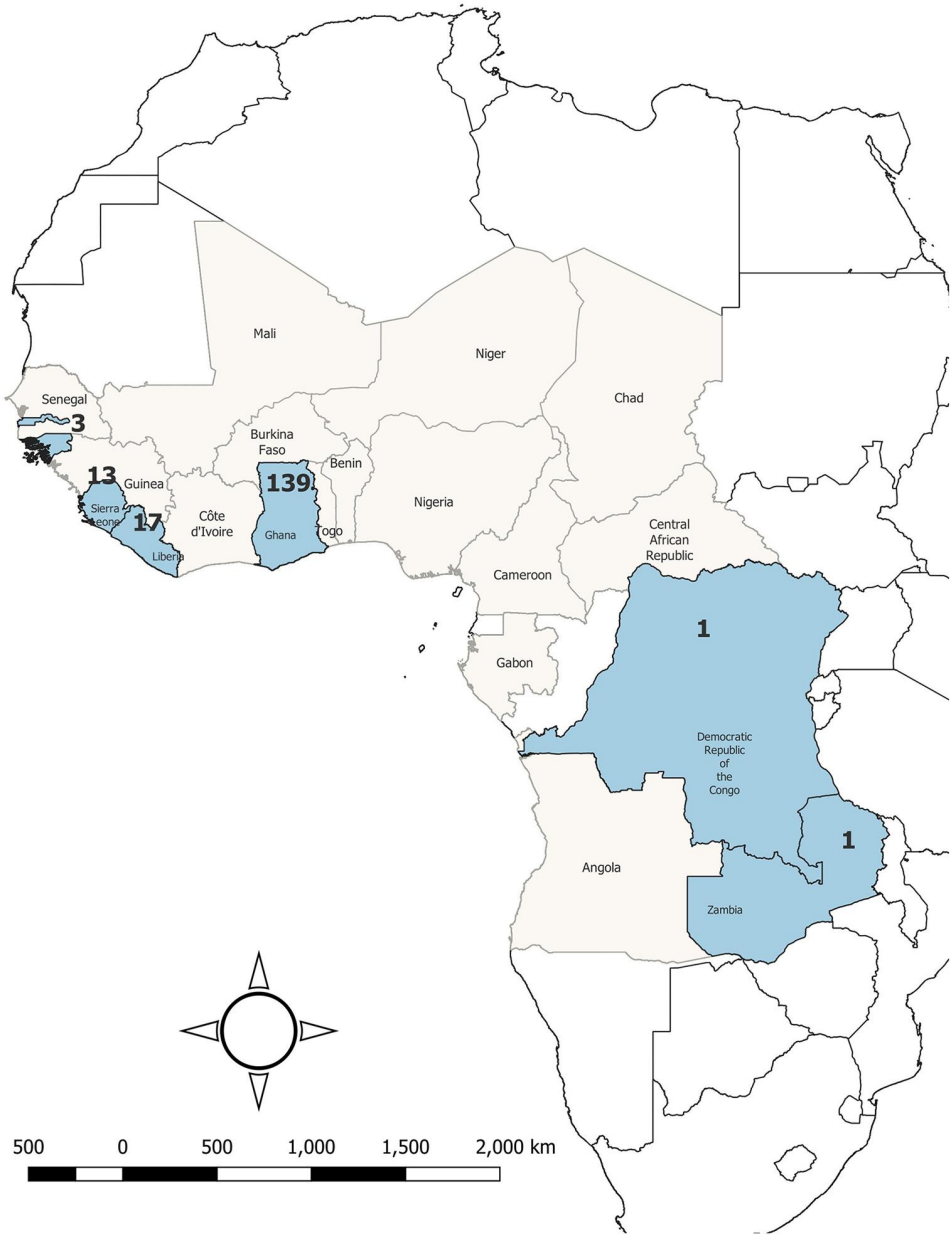
Distribution of countries involved in the GFELTP regional program and number of graduates, October 2007 to March 2024.

Since its inception, the GFELTP has been crucial in strengthening the public health workforce by training diverse health service professionals to address existing public health challenges. From October 2007 to March 2024, the program completed 15 cohorts, with two additional cohorts in training at the time of the evaluation. Most graduates (Residents who completed their degree) were from Ghana (72%), followed by Liberia (9%), The Gambia (9%), Sierra Leone (7%), and Zambia (1%). Additionally, the program trained one Francophone graduate from the Democratic Republic of the Congo (1%) and three Lusophone graduates from Guinea-Bissau (2%) (Figure 1).

**Figure 2:**
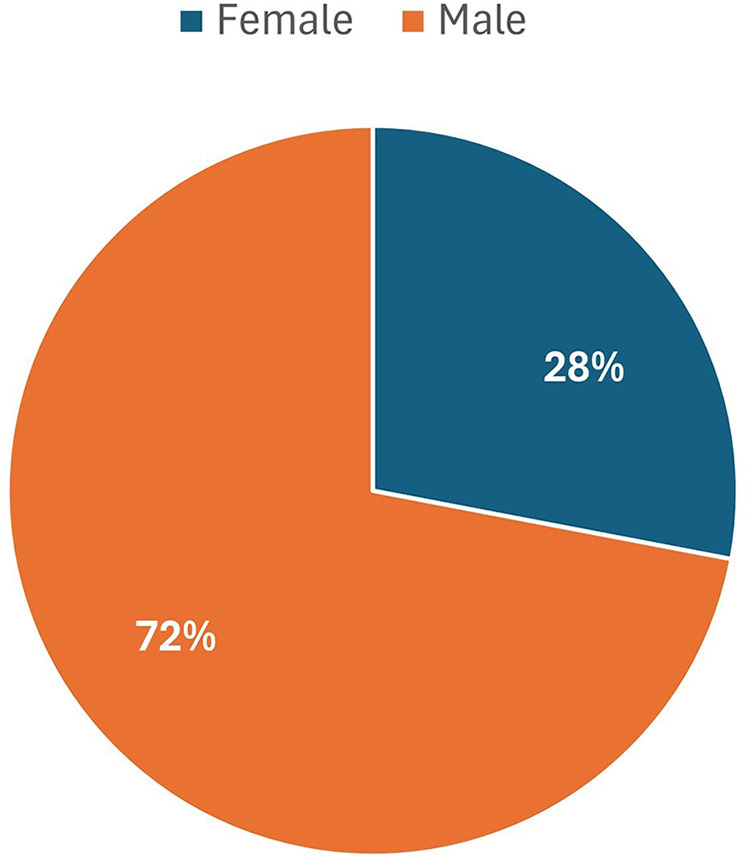
Distribution of GFELTP graduates by sex, October 2007 to March 2024.

Of the 201 residents enrolled in the FETP-Advanced program, 96% (*n* = 192) graduated; among these, 8% (*n* = 16) were enrolled in a Ph.D. program, and 4% (*n* = 8) had completed their doctoral studies. The program's graduates included officers from the human (89%), animal (8%), and environmental sectors (3%) (Figure 2, Table 1).

**Table 1:** Characteristics of GFELTP advance graduates from October 2007 to March 2024.

Variable	Frequency (*n* = 192)	Percentage (%)
Graduates’ countries of origin		
Ghana	139	72
The Gambia	18	9
Liberia	17	9
Sierra Leone	13	7
Guinea Bissau	3	2
Democratic Republic of Congo	1	1
Zambia	1	1
Track		
Human Health	171	89
Animal Health	15	8
Environmental Health	6	3

From October 2007 to March 2024, GFELTP participants contributed significantly to public health outbreak investigations. In total, residents conducted more than 200 outbreak investigations into diseases of public health importance, including measles, human rabies, influenza (H5N1), Lassa fever, and schistosomiasis. The program also conducted over 300 surveillance system evaluations and data analyses to strengthen local, national, and international disease surveillance systems. The GFELTP contributed to disseminating public health interventions to regional and global stakeholders by making over 300 scientific presentations at several local and international conferences. In addition, over 150 manuscripts have been published in national and international peer-reviewed journals. The curriculum is updated yearly to include relevant topics for the residents, either as part of the standardized curriculum or as optional short courses. GFELTP has updated its training curriculum to include modules on workplace harassment prevention, cross-border surveillance, Quantum Geographic Information System (QGIS) training, and use of reference managers such as Mendeley and Zotero (Table 2).

**Table 2:** GFELTP updated curriculum, March 2024.

YEAR 1							
Quarter 1		Quarter 2		Quarter 3		Quarter 4	
Competencies	Activities	Competencies	Activities	Competencies	Activities	Competencies	Activities
Epidemiology	Evaluation of surveillance system	Epidemiology & Fundamental Lab methods	Surveillance data analysis	Computer Technology	Computer Skills	Research Methods	Data analysis Research
YEAR 2							
Quarter 1		Quarter 2		Quarter 3		Quarter 4	
Competencies	Activities	Competencies	Activities	Competencies	Activities	Competencies	Activities
Communication and report writing	Scientific communication	Management and Leadership	Teaching Laboratory Management	Communication and report writing	Scientific communication	Public health surveillance	Outbreak investigation

### 5.2 GFELTP-advanced program staff, residents, and alumni shared the best practices

#### 5.2.1 Continuing education program

The program offered various specialized courses, including Geographic Information Systems (GIS) training for selected district and regional staff in the Greater Accra region, humanitarian emergency management and response, training for regional Emergency Operations Center (EOC) staff, and a mentorship training program.

#### 5.2.2 Career path and support for mentors

Retaining trained FETP graduates in the public sector is essential for building a resilient public health workforce and strengthening national and regional capacities to detect and respond to health threats. These skilled epidemiologists play a crucial role in disease surveillance, outbreak investigations, and overall health system strengthening. Of the 192 graduates, approximately 80%–90% continue to serve within their countries' health, agriculture, and environment ministries. Many have taken on leadership roles as program managers, district health directors, and technical advisors for multinational agencies. Others contribute as laboratory managers, FETP mentors, supervisors, or university lecturers, helping to shape the next generation of public health professionals. Additionally, some graduates have transitioned into international roles, working as FETP resident advisors, mentors, and technical experts for organizations such as AFENET, Africa CDC, WHO, and other global health institutions, extending the program's impact beyond national borders.

Field-based mentorship is a foundation of practical training in field epidemiology. To sustain mentor engagement, the program supports their costs related to field mentorship activities and, where feasible, offers networking and travel opportunities. The Dean of the University of Ghana School of Public Health has also contributed to this effort by allocating institutional funds from other projects to support residents' outbreak investigations. A program staff member further highlighted the significant impact a full-time mentor can have on the success and continuity of the advanced program.

“The intermediate program has a full-time mentor assigned to each trainee. This level of mentorship has a significant positive impact on trainee output and performance. If funding permits, exploring the option of assigning full-time mentors to the advanced program could be beneficial”.-GFELTP Staff.

#### 5.2.3 Evaluation and quality control

The program has a standardized evaluation mechanism, which includes training assessments and forms completed by residents, mentors, and supervisors on an annual basis. The Kirk-Patrick model for program evaluation is used by involving residents, mentors, and supervisors (1). Internal assessments conducted by the Ghana Teacher Education Commission (GTEC) are also in place and performed annually. GFELTP has predefined selection criteria for the recruitment of residents and mentors. A mentor is an alumnus of the program willing to support the resident throughout the training. A call is sent out to alumni to select mentors. A pool of mentors is selected and assigned based on personality traits and location. After selection, a mentors training is conducted.

#### 5.2.4 Ensure competencies acquirement

FETP is designed to develop a skilled public health workforce capable of responding to disease outbreaks, conducting surveillance, and strengthening health systems. The advanced FETP builds on this foundation by equipping professionals with higher-level competencies in epidemiological investigation, data-driven decision-making, and public health leadership.

During the first year of the training, residents are assigned to the Ghana Health Service as part of their public health practice to conduct their field activities and complete deliverables such as outbreak investigation, surveillance data analysis, surveillance system evaluation, and laboratory management. Residents originating from a country other than Ghana prepare their MPhil thesis protocols with their mentors from their original country during their stay in Ghana. The graduation rate of GFELTP was 96% for most cohorts completed, and all the residents have the required deliverables. The program organizes a peer-reviewed manuscript session led by its scientific writer. After graduation, most residents' deliverables are published in peer-reviewed scientific journals.

#### 5.2.5 Alumni network and leadership position

The GFELTP Alumni Association brings together graduates from diverse regional and cultural backgrounds, fostering a strong professional network for advancing public health. Through this network, alumni actively engage in public health initiatives, including disease outbreak response, surveillance strengthening, and community health education.

Beyond these activities, the alumni association plays a crucial role in capacity building by hosting a series of webinars to update skills and share relevant experiences. These webinars cover various topics, such as advancing public health surveillance in Ghana and advancing field epidemiology in West Africa.

As part of their continued engagement, many alumni take on mentorship roles, guiding current trainees and early-career epidemiologists. These trained mentors provide supervision, career guidance, and hands-on training opportunities, reinforcing the program's learning and leadership cycle. Through mentorship, alumni contribute to workforce development and help sustain a strong field epidemiology network across the region.

Respondents reported that during the COVID-19 pandemic response, GFELTP played a critical role by supporting the Ghana Health Services in containing the spread of the disease. The involvement of GFELTP alumni and residents in the COVID-19 pandemic response contributed to improved case investigation, case management, contact tracing, and follow-up. In 2023, the president of the Republic of Ghana recognized program graduates for their contribution to public health.

“The training programs conducted by the organization have contributed to enhancing leadership roles and outbreak response capabilities in various countries. Participants who received training in Ghana played significant roles in their respective country's COVID-19 management and surveillance efforts. While some participants may have attained leadership positions regardless, the training equipped them to function more effectively in those roles”. -Alumni.

“The training has enabled participants to identify potential outbreaks more effectively. Rather than overlooking or failing to detect outbreaks, participants can now recognize and respond to them promptly. This proactive approach enables timely mitigation measures and prevents the potential consequences of delayed responses”. -Alumni.

### 5.3 Partnership and collaboration

The GFELTP demonstrated its sustainability by adapting its offerings to meet the needs of multiple donors. The respondents reported their contributions to achieving the goals of various partner programs, such as the National Malaria Elimination Program and global health security initiatives led by USAID, CDC, and FAO, by tailoring the organization's activities and services to align with progress toward their respective objectives. The collaboration between GFELTP and its partners facilitated resource sharing and coordinated response efforts. The program has international and multilateral partners, including the Ghana Health Service, Veterinary Service Division, Food and Drugs Authority, Environmental Health and Sanitation Directorate, and the University of Ghana. Partner contributions encompass financial, human, and technical resources. The respondents reported that this improved their capability to manage public health events, and they were better prepared for new public health challenges. A respondent described the benefits of having multiple stakeholders in a program.

“The involvement of multiple stakeholders is beneficial, as it prevents reliance on a single entity and ensures diverse perspectives are considered. Engagement is also mentioned as helping to expand the program's reach and impact”. -GFELTP Staff.

Another best practice of GFELTP is that residents are not supported by a scholarship self-pay for their participation in the program. The specific amount varies depending on the year, available resources, and institutional arrangements, so a fixed fee was not provided. The key purpose of this model is to ensure sustainability through cost-sharing and to reduce dependency on external funding. This approach fosters dedication, promotes long-term sustainability, and enhances the program's impact. Additionally, it reduces reliance on external funding, ensuring the program can continue to train public health professionals even with limited scholarship availability**.**

In addition, residents and alumni highlighted the importance of maintaining the exchange program, which fosters peer learning and professional development among trainers and trainers. However, these activities are currently underfunded and require additional support.

Despite the program's successful implementation, its activities and outcomes are not sufficiently highlighted on social media or the official website. Enhancing the visibility of GFELTP through collaboration with international evaluators and technical partners such as ECDC, WHO, and academic institutions can strengthen the program's reputation, promote knowledge exchange, and ensure continuous improvement through external feedback.

## 6 Discussion

The GFELTP evaluation highlights key strengths and areas for improvement, aligning with the best global practices observed in other FETP programs. Similar evaluations of FETP programs in regions such as the United States (CDC's Epidemic Intelligence Service), Canada (CFEP), and Australia have emphasized the importance of strong institutional partnerships, sustained funding mechanisms, and post-training career pathways to ensure program sustainability and impact (8, 9).

Field Epidemiology Training Programs (FETPs) are critical workforce development initiatives to strengthen public health systems. Evaluations from other regions highlight common challenges and effective strategies for addressing them. For example, an evaluation of the Nigeria FELTP (NFELTP) revealed the importance of integrating graduates into national health structures to maintain capacity post-training (10). Similarly, the Thailand FETP has demonstrated success through strong government ownership and policy integration (11).

Over the years, FETPs have evolved into one of the most significant structures for building general public health capacity worldwide. The Ghana Field Epidemiology and Laboratory Training Program is one of the early programs in West Africa that exemplifies FETPs’ role in strengthening health systems, improving outbreak response, and sustaining the workforce. Successes, lessons learned, challenges, and implications of GFELTP are discussed for future public health training programs. The GFELTP has significantly contributed to addressing Ghana and West Africa's public health challenges by training skilled epidemiologists and laboratory specialists. This multidisciplinary training aligns with the One Health framework, which recognizes the interconnectedness of human, animal, and environmental health. By incorporating One Health principles, the GFELTP has prepared its graduates to address zoonotic diseases and other cross-sectoral health issues, a critical need in regions with frequent outbreaks of zoonotic diseases (12).

The effectiveness of FETPs can be demonstrated by assessing the competencies of the graduates and their involvement in providing public health services (13). The residents were involved in surveillance activities in Ghana and their countries of origin, resulting in positive health outcomes and improved community health. They conducted over 200 outbreak investigations on measles, human rabies, influenza (H5N1), Lassa fever, and schistosomiasis, among others. Additionally, more than 300 evaluations of surveillance systems and data analyses have been completed. These achievements align with findings from Latin American FETPs, highlighting trained epidemiologists' role in enhancing national surveillance systems (14).

These numerous achievements demonstrate that the program has successfully produced professionals who enhance existing surveillance systems and implement evidence-based interventions for public health. The program's flexibility and introduction of new curriculum modules relevant to emerging needs, such as Geographic Information Systems and cross-border surveillance, have been crucial in addressing evolving public health challenges. The Thailand FETP has similarly adapted its curriculum to incorporate modern epidemiological tools, ensuring relevance in an evolving public health landscape (11). Furthermore, its alumni network fosters continuous professional development, with several graduates holding leadership positions in their respective countries. During the COVID-19 pandemic, GFELTP graduates were directly involved in activities related to case management, contact tracing, and disease surveillance, confirming the relevance of their training in emergency preparedness and response. This is consistent with FETP alumni's role in pandemic response efforts globally, as documented in evaluations of programs in the U.S. and Canada (8, 15).

Sustainability remains the most critical consideration for FETPs, especially in resource-limited settings. GFELTP's transition toward self-support, whereby a cadre of residents supports their training, demonstrates one model of sustainability that other programs could emulate. Its partnerships with organizations such as the U.S. Centers for Disease Control and Prevention, the West African Health Organization and other international organizations have also been beneficial in securing financial and technical support for the program. Further diversification of partnerships and active engagement of local governments may enhance sustainability. Local ownership of FETPs has been described as a key ingredient in their long-term success, as observed in the institutionalization of programs such as the Philippines’ FETP and Nigeria's FELTP (10, 16). Additionally, with government support, the institutionalization of GFELTP within the University of Ghana strengthens its position as a regional training hub (14).

Mentors are essential in FELTP training as they significantly enhance trainees' learning experiences, professional development, and overall success. Qualified and experienced professionals are crucial in supporting FETP residents during their fieldwork. The program has an alumni association that mentors are recruited from. Without dedicated mentors at the Advanced level, trainees may lack the support needed to optimize their learning experiences. Dedicated mentorship has been emphasized in evaluations of U.S. and Canadian FETPs, where structured mentorship programs have led to better career progression and skill development among graduates (6, 8). Dedicated mentors can commit more time to understanding each trainee's needs, strengths, and weaknesses by providing personal guidance and regular feedback, which will help trainees identify areas for improvement.

The residents of GFELTP reported that the skills and competencies they acquired contributed to improving their country's surveillance system in collaboration with their mentors. Several FETP evaluations have documented the role of FELTPs in enhancing public health systems (8, 14, 15, 17). Other successes of the GFELTP program include participation in national and international conferences. One of the Advanced program deliverables is submitting a manuscript to a scientific peer-reviewed journal. GFELTP residents have published over 150 manuscripts in scientific journals, including the Ghana Medical Journal, BMC Public Health, JIEPH, Frontiers, and PAMJ, thereby further contributing to knowledge dissemination and public health improvement (14).

The GFELTP exchange program also enables participants to experience diverse cultural perspectives and enhance their language skills. Each program involves participants traveling to other countries for field activities and working with teams from diverse backgrounds. The GFELTP and the Burkina Faso FELTP conducted two simulation exercises across the border in 2020 and 2021 as part of regional collaboration. Similar cross-border initiatives have been undertaken in East Africa and Latin America to enhance regional preparedness, as documented in evaluations by PAHO and WHO (14, 19). Ensuring the continuity of exchange activities will require targeted resource mobilization and integration into the program's long-term strategy. Exchange visits not only enhance skills but also foster cross-institutional collaboration. Strengthening these exchange programs will align with global best practices and improve regional health security. Improved communication through digital platforms is vital for raising awareness of the program's impact. In addition to improving digital visibility, ensuring adequate network connectivity in field sites is critical for effective adoption of digital tools. Equally important is the need to strengthen digital literacy among community leads and field officers to foster familiarity, confidence, and sustainability in platform use. Without concurrent investments in connectivity and user education, digital initiatives risk underutilization despite their potential value.

## 7 Limitations

The study did not include critical stakeholders such as field supervisors, government health officials, and international partners who could have provided valuable insights into the program's impact and alignment with national and global public health priorities. The evaluation did not involve visits to field sites where residents conducted their work. Such observations could have provided a more comprehensive understanding of the program's practical challenges and achievements in real-world settings. The evaluation did not benchmark GFELTP against other regional or global FETPs, limiting the ability to contextualize its performance relative to similar programs. As the time and resources allocated to this assessment were extremely limited, our methods could not include most of the program's beneficiaries, graduates' field supervisors, and stakeholders supporting the program.

## 8 Conclusion

The GFELTP assessment highlights the program's significant contributions to public health capacity building and its effectiveness in training epidemiologists and public health professionals by equipping them with the skills, knowledge, and networks necessary to address current and future public health challenges in Ghana and West Africa. While there are areas for improvement, the GFELTP is a foundation for advancing epidemiological capacity building and improving population health outcomes in West African countries. With an entirely Ghanaian staff, the diversity of partners, the self-sufficiency of residents, and the development of an income-generating strategy and institutionalization of the program at the University of Ghana are assets that have contributed to the program's sustainability. Continued investment and support for the GFELTP are essential to sustain and enhance its impact in West Africa. Dedicated mentors provide real-time feedback on residents' deliverables, improving technical skills such as data analysis and outbreak investigation. For the program, it is crucial to maintain the exchange program activities, and it is essential to find additional support to facilitate this collaboration. There is a need to increase the program's visibility on social media and the website, as it is doing a lot but is not being showcased.

## Data Availability

The original contributions presented in the study are included in the article/Supplementary Material, further inquiries can be directed to the corresponding author.
